# Knowledge, Attitudes, and Preventive Practices Regarding Tuberculosis Among Healthcare Workers and Patients in India: A Mixed-Method Study

**DOI:** 10.7759/cureus.56368

**Published:** 2024-03-18

**Authors:** Jeel Shihora, Naresh C Damor, Alpesh Parmar, Nikhil Pankaj, Yogesh Murugan

**Affiliations:** 1 Preventive Medicine, Shri M. P. Shah Medical College, Jamnagar, IND; 2 Community Medicine, Shri M. P. Shah Medical College, Jamnagar, IND; 3 Public Health, Shri M. P. Shah Medical College, Jamnagar, IND; 4 Pulmonary Medicine, Shri M. P. Shah Medical College, Jamnagar, IND; 5 Family Medicine, Guru Gobind Singh Government Hospital, Jamnagar, IND

**Keywords:** patients, healthcare workers, attitudes, knowledge, prevention, tuberculosis

## Abstract

Background: Tuberculosis (TB) remains a major public health challenge in India. Healthcare workers (HCWs) and TB patients are critical to disease transmission and prevention. This study evaluated and compared knowledge, attitudes, and practices related to TB infection control.

Materials and methods: This was a mixed-method study that included a cross-sectional survey conducted among 167 HCWs and 346 patients with TB at tertiary hospitals in western Gujarat using a validated questionnaire. Additionally, 20 HCWs and 20 patients were interviewed to gain qualitative insights. Between-group analyses were performed, and multivariate regressions identified predictors of knowledge and compliance, while thematic analysis explored qualitative insights.

Results: A total of 142/167 (85.0%) HCWs had good knowledge, whereas 208/346 (60.1%) patients had good knowledge. A total of 151/167 (90.4%) HCWs had positive attitudes, whereas 242/346 (69.9%) patients had positive attitudes. A total of 159/167 (95.2%) HCWs practiced good preventive behaviors, whereas 225/346 (65.0%) patients did. HCWs demonstrated significantly higher mean knowledge scores (9.2 vs. 7.1, p<0.001) and higher median attitude scores (ranging from 5 with IQR 4-5 to 5 with IQR 5-5) compared to patients (ranging from 4 with IQR 3-5 to 5 with IQR 4-5) across all attitude statements assessed using the Likert scale (p<0.001). Being an HCW was associated with good knowledge (adjusted odds ratio (AOR) 2.5, 95% CI 1.5-4.1), positive attitudes (AOR 2.2, 95% CI 1.4-3.6), and good practices (AOR 3.1, 95% CI 1.8-5.2). The qualitative themes highlighted the need for accessible education, clear communication, adequate resourcing, and personal responsibility.

Conclusion: This study identified gaps in TB prevention knowledge and practices among patients compared to those among HCWs in India. Tailored educational initiatives, optimized health communication, improved infrastructure, and accessible messaging can help individuals assimilate appropriate infection control behaviors among healthcare system actors and communities toward ending TB.

## Introduction

Tuberculosis (TB) remains a major global public health concern, with an estimated 10 million cases and 1.4 million deaths occurring in 2019 [1]. India has the highest burden, accounting for more than a quarter of new cases annually [2]. Thus, maintaining robust infection control and prevention is imperative.

Healthcare workers (HCWs) serve as an interface between health systems and communities. Their knowledge, attitudes, and compliance with preventive precautions significantly impact disease transmission risks and mitigation [3]. However, evidence indicates sustained gaps in TB awareness and preventive practices among HCWs in high-burden countries [4].

Similarly, patients’ infection control behavior also modulates exposure in clinical and community contexts [5]. However, social, economic, and healthcare access barriers can impede patients’ comprehension and adoption of TB prevention strategies [6].

Mixed findings exist regarding specific sociodemographic variables and their association with TB prevention knowledge and practices among HCWs and patients in India [7,8]. Additionally, few studies have concurrently evaluated and compared both groups using a mixed-methods approach.

This study aimed to assess and contrast TB-related knowledge, attitudes, and practices among HCWs and patients at tertiary hospitals in an Indian city through a cross-sectional survey and qualitative interviews. The findings are intended to highlight specific awareness and compliance gaps requiring tailored policy and education interventions to mitigate TB spread.

## Materials and methods

Study design and settings

This study employed a mixed-method approach, combining both quantitative and qualitative methods. A cross-sectional survey was conducted to assess the knowledge, attitudes, and preventive practices of HCWs and TB patients visiting tertiary care centers. Additionally, qualitative interviews were conducted to gain deeper insights into the perspectives and experiences of participants regarding infection prevention and control practices.

Sample size justification

Although the sample size was not primarily determined based on statistical power, a post hoc power analysis was conducted to ensure that the study had sufficient power to detect significant differences between HCWs and patients. With a sample size of 167 HCWs and 346 patients with TB, assuming an alpha level of 0.05 and a medium effect size (Cohen's d = 0.5), the study achieved a power of approximately 0.99. This indicates that the sample size was adequate to detect significant differences between the two groups.

Qualitative data saturation

For the qualitative component of the study, 20 HCWs and 20 patients were interviewed. This sample size is consistent with recommendations for achieving data saturation in qualitative research. Data saturation was reached when no new themes or information emerged from additional interviews, ensuring that the qualitative findings were comprehensive and representative of the participants' perspectives [9].

Sampling technique

Selection of HCWs

Among the 167 HCWs included in the study, the distribution of designations was as follows: doctors: 80 (47.9%), nurses: 60 (35.9%), and other HCWs (e.g., laboratory technicians, pharmacists). Twenty-seven (16.2%) HCWs were selected using a stratified random sampling technique. First, the HCWs were stratified based on their designations (doctors, nurses, and other HCWs) to ensure adequate representation of each group. Then, within each stratum, participants were randomly selected using a computer-generated random number list. This approach ensures that the sample is representative of the different categories of HCWs working in tertiary care centers. Using a random number generator, a simple random technique was used to randomly select participants from among patients with TB visiting the outpatient clinic of pulmonary medicine in a tertiary care hospital, ensuring that each eligible patient had an equal chance of being included in the study.

For the qualitative component, a purposive sampling technique was used to select participants until data saturation was reached. A minimum of 20 HCWs and 20 patients were interviewed.

Inclusion and exclusion criteria for HCWs

Inclusion Criteria

Healthcare professionals (doctors, nurses, laboratory technicians, pharmacists, etc.) currently employed at tertiary care centers and HCWs who have direct or indirect contact with patients with TB during their duties, HCWs who had been employed at the tertiary care center for at least six months, and willingness to participate in the study and provide informed consent.

Exclusion Criteria

HCWs who are not involved in the care or management of patients with TB, HCWs who are currently on long-term leave or who have plans to leave the institution within the study period, the cognitive or mental impairments of HCWs may affect their ability to understand and respond to the questionnaire or interviews, and HCWs who declined to participate in the study.

Inclusion and exclusion criteria for TB patients

Inclusion Criteria

Patients aged 18 years or older were diagnosed with active pulmonary TB (bacteriologically confirmed or clinically diagnosed) within the past six months, patients were registered for treatment at the outpatient clinic of the pulmonary medicine department at the tertiary care center, patients who were capable of providing informed consent and responding to the questionnaire or interviews, and willingness to participate in the study and provide informed consent.

Exclusion Criteria

Patients with extrapulmonary TB or other forms of TB not affecting the lungs, severe comorbidities or cognitive impairments that may affect patients’ ability to understand and respond to the questionnaire or interviews, patients who were critically ill or hospitalized at the time of data collection, patients who declined to participate in the study, and patients who were unable to communicate effectively in the language(s) used for data collection.

Data collection tool

A structured questionnaire was developed to assess knowledge, attitudes, and preventive practices among HCWs and patients. The questionnaire was designed based on relevant literature and guidelines and was piloted for clarity and comprehensibility. The survey included sections on demographics, knowledge questions (general, HCW-specific, and patient-specific), attitude statements (Likert scale), and preventive practices (general, HCW-specific, and patient-specific) [10,11].

For the qualitative component, semi-structured interview guides were developed separately for HCWs and patients. The guides included open-ended questions exploring themes such as the importance of education and awareness, the role of media, challenges in maintaining preventive practices, the need for institutional support, and personal responsibility.

Validity and reliability of the questionnaire

This study utilized a structured questionnaire to assess the knowledge, attitudes, and preventive practices related to TB among HCWs and patients. The questionnaire was developed based on an extensive review of relevant literature and guidelines from reputable sources such as the World Health Organization (WHO) and national TB control programs [10,11].

Validity

To ensure the validity of the questionnaire, several measures were taken.

Content validity: The questionnaire items were carefully designed to cover all relevant domains of TB prevention knowledge, attitudes, and practices and were aligned with established guidelines and recommendations.

Face validity: The initial draft of the questionnaire was reviewed by a panel of experts, including experienced TB physicians, epidemiologists, and infection control specialists. Their feedback was incorporated to improve the clarity, relevance, and appropriateness of the items.

Construct validity: The questionnaire was pilot-tested on a small sample of HCWs and patients to assess whether the items accurately measured the intended constructs. Exploratory factor analysis was performed to examine the underlying factor structure and ensure that the items loaded appropriately on the respective domains.

Reliability

The reliability of the questionnaire was evaluated using the following approaches.

Internal consistency: Cronbach's alpha coefficient was calculated to assess the internal consistency and reliability of the knowledge, attitude, and practice sections of the questionnaire. A Cronbach's alpha value of 0.7 or higher was considered acceptable, indicating good internal consistency.

Test-retest reliability: A subset of participants (both HCWs and patients) completed the questionnaire twice, with an interval of 2-4 weeks between the two administrations. Test-retest reliability was assessed by calculating the intraclass correlation coefficient (ICC) or Pearson's correlation coefficient for the total scores and individual domain scores. An ICC or correlation coefficient of 0.7 or higher was considered acceptable, indicating good test-retest reliability.

The findings from the validity and reliability assessments were used to refine and finalize the questionnaire before its implementation in the main study. Any items with poor performance or ambiguity were revised or removed to ensure the overall robustness of the instrument.

Procedure

The data collectors were trained on the study protocol, questionnaire administration, and interview techniques. Potential participants (HCWs and patients) at tertiary care centers were approached, and informed consent was obtained. The structured questionnaire was administered to participants, and completed questionnaires were collected. Qualitative interviews were conducted with selected HCWs and patients until data saturation was reached. The collected data were compiled and analyzed.

Operational definitions

Good knowledge is defined as scoring above a predetermined cutoff point (≥80%) on the knowledge questions. Positive attitude is achieving a mean score of ≥4 on the Likert scale for attitude statements. Good practices are adhering to a set of predetermined preventive practices (regular handwashing, wearing masks, maintaining physical distance).

Data analysis

Quantitative data were analyzed using descriptive statistics (frequencies, percentages, means, standard deviations) and inferential statistics (chi-square test, Mann-Whitney U test, logistic regression) to compare knowledge, attitudes, and practices between HCWs and patients. Logistic regression was used to identify factors associated with good knowledge, positive attitudes, and good practices. A p-value < 0.05 was considered to indicate statistical significance.

The qualitative data were transcribed verbatim and analyzed via thematic analysis. The data were coded and categorized into themes and subthemes, and relevant participant quotes were used to support the findings. The qualitative findings were used to complement and enrich the quantitative results.

Ethical consideration

The study was approved by the Institutional Ethics Committee (REF No: 17/01/2023). Written informed consent was obtained from all participants. Participant anonymity was maintained using unique identifiers.

## Results

Demographic characteristics

The study included a total of 167 HCWs and 346 patients with TB. The mean age of the HCWs was 35.6 ± 8.2 years, while the mean age of the patients was 48.3 ± 12.5 years. Among the HCWs, 100 (59.9%) were female, and 67 (40.1%) were male. In the patient group, 156 (45.1%) were female, and 190 (54.9%) were male. The majority of HCWs had a college education (59%) or postgraduate degree (30%), while 39.9% of patients had a high school education, and 50% had a college education. Most HCWs (58%) and patients (54.9%) had a monthly income between 10,000 rupees and 50,000 rupees. The HCW group consisted of 80 (47.9%) doctors, 60 (35.9%) nurses, and 27 (16.2%) other HCWs. Among the patients, 208 (60.1%) were employed, and 138 (39.9%) were unemployed. The predominant religion was Hinduism in both HCWs (60%) and patients (65%), followed by Islam (29% in HCWs and 30.1% in patients) (Table 1).

**Table 1:** Demographic characteristics of study participants

Characteristic	HCWs (n = 167)	Patients (n = 346)
Age (mean ± SD) in years	35.6 ± 8.2	48.3 ± 12.5
Gender		
- Male	67 (40.1%)	190 (54.9%)
- Female	100 (59.9%)	156 (45.1%)
Education		
- High school	17 (11%)	138 (39.9%)
- College	99 (59%)	173 (50.0%)
- Postgraduate	51 (30%)	35 (10.1%)
Income		
- <10000	21 (13%)	104 (30.1%)
10000-50000	97 (58%)	190 (54.9%)
- >50000	49 (29%)	52 (15.0%)
Occupation		
- Doctors	80 (47.9%)	-
- Nurses	60 (35.9%)	-
- Other HCWs	27 (16.2%)	-
- Employed	-	208 (60.1%)
- Unemployed	-	138 (39.9%)
Religion		
- Hinduism	101 (60%)	225 (65.0%)
- Islam	49 (29%)	104 (30.1%)
- Others	17 (11%)	17 (4.9%)

Knowledge scores

The overall knowledge score was significantly greater among HCWs (9.2 ± 0.8) than among patients (7.1 ± 1.5) (p < 0.001). Almost all HCWs were aware of the importance of infection prevention (98.8%), common modes of transmission (97%), the role of personal protective equipment (PPE) (100%), and the importance of hand hygiene (100%). Among patients, 85% were aware of the importance of infection prevention, 80.1% knew common modes of transmission, 69.9% understood the role of PPE, and 89.9% were aware of the importance of hand hygiene. HCW-specific knowledge questions revealed that 95.8% knew the correct procedure for donning and doffing PPE, 98.2% understood the importance of proper waste disposal, 95.2% knew the indications for using different types of masks, and 97% were aware of the need for regular training and updates. Patient-specific knowledge questions showed that 80.1% knew when to seek medical attention for symptoms, 91.9% understood the importance of following medical advice, 74.9% were aware of the need to disclose relevant information to HCWs, and 87% knew the importance of completing the prescribed treatment (Table 2).

**Table 2:** Knowledge scores of HCWs and patients with tuberculosis *p < 0.05, **p < 0.01, ***p < 0.001 HCWs: Healthcare workers; PPE: Personal protective equipment; TB: Tuberculosis

Knowledge questions	HCWs (n = 167)	Patients with TB (n = 346)
General knowledge		
- Aware of the importance of infection prevention	165 (98.8%)	294 (85.0%)
- Know common modes of transmission	162 (97.0%)	277 (80.1%)
- Understand the role of personal protective equipment	167 (100%)	242 (69.9%)
- Aware of the importance of hand hygiene	167 (100%)	311 (89.9%)
HCW-specific knowledge		
- Know the correct procedure for donning and doffing PPE	160 (95.8%)	-
- Understand the importance of proper waste disposal	164 (98.2%)	-
- Know the indications for using different types of masks	159 (95.2%)	-
- Aware of the need for regular training and updates	162 (97.0%)	-
Patient-specific knowledge		
- Know when to seek medical attention for symptoms	-	277 (80.1%)
- Understand the importance of following medical advice	-	318 (91.9%)
- Aware of the need to disclose relevant information to HCWs	-	259 (74.9%)
- Know the importance of completing the prescribed treatment	-	301 (87.0%)
Overall knowledge score (mean ± SD)	9.2 ± 0.8	7.1 ± 1.5
p-value	<0.001 **	

Attitudes toward preventive practices

Table 3 presents the attitudes toward preventive practices among HCWs and patients, assessed using a 5-point Likert scale ranging from 1 (strongly disagree) to 5 (strongly agree). The table reports the median and interquartile range (IQR) of the Likert scale responses for each attitude statement.

**Table 3:** Attitudes toward preventive practices (Likert scale: 1 - strongly disagree to 5 - strongly agree) *p < 0.05, **p < 0.01, ***p < 0.001 HCWs: Healthcare workers; IQR: Interquartile range

Statement	Median (IQR) (HCWs)	Median (IQR) (patients with tuberculosis)	p-value
1. Handwashing is important to prevent infections	5 (4-5)	4 (3-5)	<0.001**
2. Wearing masks helps reduce the spread of infections	5 (4-5)	4 (3-5)	<0.001**
3. Vaccination is crucial for disease prevention	5 (4-5)	4 (3-5)	<0.001**
4. Regular cleaning and disinfection of surfaces is essential	5 (5-5)	4 (3-5)	<0.001**
5. Proper waste disposal is crucial in healthcare settings	5 (4-5)	4 (3-5)	<0.001**
6. Personal protective equipment (PPE) is essential for HCWs	5 (5-5)	5 (4-5)	<0.001**
7. Patients should disclose their infection status to HCWs	5 (4-5)	4 (3-5)	<0.001**
8. Isolation and quarantine measures are important during outbreaks	5 (4-5)	4 (3-5)	<0.001**
9. Continuous education and training are necessary for effective prevention	5 (4-5)	4 (3-5)	<0.001**
10. Collaboration between HCWs and patients is key to preventing infections	5 (4-5)	4 (4-5)	<0.001**

The results from the Mann-Whitney U test indicate that HCWs had significantly higher median scores compared to patients for all attitude statements (p < 0.001), suggesting more positive attitudes toward preventive practices among HCWs.

For instance, the median score for the statement "regular cleaning and disinfection of surfaces are essential" was 5 (IQR: 5-5) among HCWs, which is significantly higher than the median score of 4 (IQR: 3-5) among patients. Similarly, the median score for the statement "personal protective equipment (PPE) is essential for HCWs" was 5 (IQR: 5-5) among HCWs and 5 (IQR: 4-5) among patients, with a statistically significant difference between the two groups.

Among HCWs, the statements with the highest median scores were "regular cleaning and disinfection of surfaces are essential" and "personal protective equipment (PPE) is essential for HCWs," both with a median of 5 (IQR: 5-5). For patients, the statement with the highest median score was "personal protective equipment (PPE) is essential for HCWs," with a median of 5 (IQR: 4-5).

The use of median, IQR, and the Mann-Whitney U test is more appropriate for analyzing and comparing ordinal data, such as Likert scale responses, between groups. These non-parametric methods do not make assumptions about the underlying distribution of the data, which is often violated in the case of ordinal data.

Preventive practices

HCWs demonstrated significantly better preventive practices than patients (p < 0.001) for general preventive measures and vaccination status. Regular handwashing with soap and water or hand sanitizer was practiced by 95.2% of HCWs and 69.9% of patients. Wearing masks in crowded places was followed by 90.4% of HCWs and 60.1% of patients. Maintaining physical distance from others was observed by 85% of HCWs and 54.9% of patients. Covering the mouth and nose when coughing or sneezing was practiced by 98.2% of HCWs and 80.1% of patients. Among HCWs, 98.8% reported proper use of PPE, 97% performed regular cleaning and disinfection of work surfaces, 95.2% followed safe handling and disposal of infectious waste, and 88.6% participated in infection control training and education. Patient-specific preventive practices revealed that 65% of patients disclosed their infection status to healthcare providers, 74.9% adhered to prescribed treatment regimens, 60.1% followed isolation and quarantine guidelines when necessary, and 80.1% sought medical attention for symptoms suggestive of infections. The COVID-19 vaccination rate was significantly greater among HCWs (98.8%) than among patients (74.9%) (p < 0.001) (Table 4).

**Table 4:** Preventive practices used by HCWs and patients with tuberculosis *p < 0.05, **p < 0.01, ***p < 0.001 HCWs: Healthcare workers

Preventive practices	HCWs (n = 167)	Patients (n = 346)	p-value
General preventive practices			
- Regular handwashing with soap and water or hand sanitizer	159 (95.2%)	242 (69.9%)	<0.001 **
- Wearing masks in crowded places	151 (90.4%)	208 (60.1%)	<0.001 **
- Maintaining physical distance from others	142 (85.0%)	190 (54.9%)	<0.001 **
- Covering mouth and nose when coughing or sneezing	164 (98.2%)	277 (80.1%)	<0.001 **
HCW-specific preventive practices			
- Proper use of personal protective equipment (PPE)	165 (98.8%)	-	-
- Regular cleaning and disinfection of work surfaces	162 (97.0%)	-	-
- Safe handling and disposal of infectious waste	159 (95.2%)	-	-
- Participation in infection control training and education	148 (88.6%)	-	-
Patient-specific preventive practices			
- Disclosure of infection status to healthcare providers	-	225 (65.0%)	-
- Adherence to prescribed treatment regimens	-	259 (74.9%)	-
- Following isolation and quarantine guidelines when necessary	-	208 (60.1%)	-
- Seeking medical attention for symptoms suggestive of infections	-	277 (80.1%)	-
Vaccination status			
Received recommended vaccination (COVID-19)	165 (98.8%)	259 (74.9%)	<0.001 **

Table 5 illustrates the level of knowledge, attitudes, and practices of HCWs and patients with TB. The results show that a greater percentage of HCWs had good knowledge (85.0%), positive attitudes (90.4%), and good practices (95.2%) than patients (60.1%, 69.9%, and 65.0%, respectively). The p-values indicate that the differences between HCWs and patients are statistically significant for all three variables (p < 0.001).

**Table 5:** Frequencies and percentage of HCWs and patients with good knowledge, positive attitudes, and good practices *p < 0.05, **p < 0.01, ***p < 0.001 HCWs: Healthcare workers

Variables	HCWs (n = 167)	Patients (n = 346)	p-value
Good knowledge			
- Yes	142 (85.0%)	208 (60.1%)	<0.001 **
- No	25 (15.0%)	138 (39.9%)	
Positive attitude			
- Yes	151 (90.4%)	242 (69.9%)	<0.001 **
- No	16 (9.6%)	104 (30.1%)	
Good practices			
- Yes	159 (95.2%)	225 (65.0%)	<0.001 **
- No	8 (4.8%)	121 (35.0%)	

Factors associated with good knowledge, positive attitudes, and good practices

Multivariate logistic regression analysis revealed that higher education levels (college and postgraduate) were significantly associated with good knowledge, positive attitudes, and good practices. Being a HCW was significantly more strongly associated with good knowledge (adjusted odds ratio (AOR): 2.5, 95% CI: 1.5-4.1), positive attitudes (AOR: 2.2, 95% CI: 1.4-3.6), and good practices (AOR: 3.1, 95% CI: 1.8-5.2) among HCWs than among non-HCWs. Higher-income levels (>50,000 rupees) were significantly associated with good knowledge (AOR: 2.0, 95% CI: 1.2-3.4), positive attitudes (AOR: 1.7, 95% CI: 1.0-2.8), and good practices (AOR: 1.9, 95% CI: 1.1-3.2). Female gender was significantly associated with positive attitudes (AOR: 1.6, 95% CI: 1.1-2.4) (Table 6).

**Table 6:** Association between good knowledge, positive attitude, and good practices and sociodemographic characteristics (multivariate logistic regression) *p < 0.05, **p < 0.01, ***p < 0.001 HCWs: Healthcare workers; AOR: adjusted odds ratio; CI: confidence interval

Sociodemographic characteristics	Good knowledge	Positive attitude	Good practices
	AOR (95% CI)	AOR (95% CI)	AOR (95% CI)
Age			
- 18-30 years	1 (Reference)	1 (Reference)	1 (Reference)
- 31-45 years	1.5 (0.9-2.4)	1.2 (0.8-1.9)	1.3 (0.8-2.1)
- 46-60 years	1.1 (0.7-1.8)	1.4 (0.9-2.2)	0.9 (0.6-1.5)
- >60 years	0.8 (0.5-1.4)	1.1 (0.7-1.8)	0.7 (0.4-1.2)
Gender			
- Male	1 (Reference)	1 (Reference)	1 (Reference)
- Female	1.4 (0.9-2.1)	1.6 (1.1-2.4)*	1.2 (0.8-1.8)
Education			
- High school or less	1 (Reference)	1 (Reference)	1 (Reference)
- College	2.1 (1.3-3.4)**	1.5 (0.9-2.3)	1.7 (1.1-2.7)*
- Postgraduate	2.8 (1.6-4.8)***	1.9 (1.1-3.1)*	2.2 (1.3-3.7)**
Occupation			
- HCWs	2.5 (1.5-4.1)***	2.2 (1.4-3.6)**	3.1 (1.8-5.2)***
- Non-HCWs	1 (Reference)	1 (Reference)	1 (Reference)
Income			
- <10000	1 (Reference)	1 (Reference)	1 (Reference)
10000-50000	1.6 (1.0-2.6)*	1.4 (0.9-2.2)	1.5 (0.9-2.4)
- >50000	2.0 (1.2-3.4)**	1.7 (1.0-2.8)*	1.9 (1.1-3.2)*

Qualitative findings

The qualitative interviews revealed five main themes: the importance of education and awareness, the role of media in promoting preventive practices, challenges in maintaining preventive practices, the need for institutional support and resources, and personal responsibility. HCWs emphasized the importance of continuous training and updates, while patients expressed the need for accessible information and clarity in communication. Both groups acknowledged the positive influence of media in promoting preventive practices but also cautioned against misinformation. Time constraints, discomfort, forgetfulness, and inconvenience were identified as challenges in maintaining preventive practices. HCWs highlighted the need for adequate supplies and staffing to ensure adherence to infection prevention protocols. Patients emphasized the importance of individual actions and encouraged others to follow preventive measures (Table 7).

**Table 7:** Themes, subthemes, and participant phrases from qualitative interviews HCWs: Healthcare workers

Themes and subthemes	HCWs	Patients
1. Importance of education and awareness	1.1. Continuous training and updates "Regular training sessions help us stay updated with the latest infection prevention guidelines." 1.2. Patient education "It is crucial to educate patients about the importance of following preventive measures to reduce the spread of infections."	Accessible information "I wish there were more easily accessible resources to learn about preventing infections." Clarity in communication "Sometimes, the information provided by healthcare workers is too complex. It would be helpful if they could explain things in simpler terms."
2. Role of media in promoting preventive practices	2.1. Positive influence "Media campaigns can play a significant role in promoting good hygiene practices among the general public." 2.2. Misinformation "We need to be cautious about the spread of misinformation through media channels, which can lead to confusion and mistrust."	2.1. Awareness through media "I learned about the importance of wearing masks and maintaining physical distance through TV advertisements and social media posts." 2.2. Conflicting messages "Sometimes, the information provided by different media sources is contradictory, making it difficult to know what to believe."
3. Challenges in maintaining preventive practices	3.1. Time constraints "With the heavy workload, it can be challenging to find time for regular hand hygiene and proper use of PPE." 3.2. Discomfort "Wearing PPE for extended periods can be uncomfortable and lead to fatigue."	3.1. Forgetfulness "I sometimes forget to wash my hands or wear a mask, especially when I'm in a hurry." 3.2. Inconvenience "Following all the preventive measures can be inconvenient at times, like having to constantly sanitize my hands or maintain distance from others."
4. Need for institutional support and resources	4.1. Adequate supplies "It is important for the hospital administration to ensure an uninterrupted supply of PPE, hand sanitizers, and other necessary resources." 4.2. Staffing "Adequate staffing is crucial to ensure that we have enough time to follow infection prevention protocols while providing quality patient care."	-
5. Personal responsibility	-	5.1. Individual actions "I believe that every person has a responsibility to follow preventive measures to protect themselves and others." 5.2. Encouraging others "I try to encourage my family and friends to follow good hygiene practices and stay up-to-date with their vaccinations."

In summary, this study highlights the significant differences in knowledge, attitudes, and preventive practices between HCWs and patients with TB. The findings underscore the importance of targeted interventions to improve knowledge, attitudes, and practices among patients as well as the need for continuous training and support for HCWs. These qualitative insights provide valuable information for designing effective infection prevention and control strategies that address the specific needs and challenges faced by HCWs and patients.

## Discussion

This mixed-methods study revealed significant differences in knowledge, attitudes, and practices related to TB prevention between HCWs and patients with TB.

Overall, compared with patients, HCWs had better knowledge, more positive attitudes, and greater adherence to preventive practices. The mean knowledge score was significantly greater among HCWs (9.2 ± 0.8 vs. 7.1 ± 1.5; p<0.001), indicating good awareness about TB transmission, the use of protection, and the importance of precautions such as hand hygiene [12]. Nearly all HCWs correctly answered job-specific questions, reflecting training. In contrast, knowledge gaps existed among patients, highlighting education needs.

Likewise, HCWs expressed stronger agreement with preventive measure statements, scoring 4.5-4.9 out of 5. The highest level of agreement related to environmental hygiene and personal protection equipment was aligned with workplace policies. Patients showed lower average agreement scores despite acknowledging their importance [13].

In practice, more than 90% of HCWs followed hand, respiratory hygiene, and mask recommendations, which is attributable to infection control training and monitoring [14]. Patient adherence was lower, likely due to forgetfulness, stigma, and financial constraints [15].

Superior HCW knowledge and practices mirror studies globally [16]. Qualitative insights revealed contributing factors. HCWs emphasized the role of regular education, adequate resources, and manageable workloads in enabling adherence. Patients expressed needing simplified, accessible information, with misconceptions spreading otherwise.

Regression analysis identified predictors of knowledge, attitudes, and practices. Higher education, occupation in healthcare, and income were associated with better scores, consistent with the literature [17]. Unexpectedly, the female gender predicted greater awareness, contrasting with the findings of other studies [18] but aligning with those of other studies [19]. Further exploration is warranted.

Study implications include the need for tailored patient education, HCW communication skills training, institutional resourcing and monitoring to enable sustainable adherence [18], and coordinated public messaging to motivate behavioral change [20-23].

Limitations

This study has several limitations that should be considered. The single-center design makes the findings less generalizable to other settings. Self-report questionnaire responses introduce the possibility of response bias. The cross-sectional rather than the interventional design limits determinations of causality. The perspectives captured through qualitative interviews, while reaching saturation, included only 20 HCWs and 20 patients, and as knowledge and practices evolve, assessments at a single time point have constraints.

Recommendations

Based on the study's findings and limitations, the following recommendations are made: Educational programs to address gaps in TB knowledge among patients regarding transmission, use of precautions, and preventive measures. Improved HCW communication through counseling will increase patient comprehension of TB prevention information. Adequate institutional resources and staffing are needed to enable monitoring and training on TB infection control protocols. Consistent public health messaging regarding TB precautions to mitigate misinformation and improve compliance and further studies across multiple centers evaluating the impacts of awareness campaigns and behavior change interventions on TB prevention over time. Additional research comparing practices across high- and low-TB burden settings is needed to identify contextual barriers and facilitators.

## Conclusions

This study identified crucial gaps in knowledge of TB prevention and practices among HCWs and patients in tertiary care centers in India. Tailored educational approaches, strengthened healthcare communication, adequate resourcing for infection control, and consistent public messaging are vital to curbing TB transmission. Regular evaluations and implementation research on awareness-raising effectiveness are warranted to assess preventive behaviors among health system actors and at-risk communities.
